# SCR-Net: A Dual-Channel Water Body Extraction Model Based on Multi-Spectral Remote Sensing Imagery—A Case Study of Daihai Lake, China

**DOI:** 10.3390/s25030763

**Published:** 2025-01-27

**Authors:** Zhi Weng, Qiyan Li, Zhiqiang Zheng, Lixin Wang

**Affiliations:** 1 School of Electronic Information Engineering, Inner Mongolia University, Hohhot 010021, China; wzhi@imu.edu.cn (Z.W.); liqiyan716@163.com (Q.L.); 2 Collaborative Innovation Center for Grassland Ecological Security, Ministry of Education of China, Hohhot 010021, China; lx_wimu@imu.edu.cn; 3 School of Ecology and Environment, Inner Mongolia University, Hohhot 010021, China

**Keywords:** lake area, remote sensing, deep learning, semantic segmentation, ecology

## Abstract

Monitoring changes in lake area using remote sensing imagery and artificial intelligence algorithms is essential for assessing regional ecological balance. However, most current semantic segmentation models primarily rely on the visible light spectrum for feature extraction, which fails to fully utilize the multi-spectral characteristics of remote sensing images. Therefore, this leads to issues such as blurred segmentation of lake boundaries in the imagery, the loss of small water body targets, and incorrect classification of water bodies. Additionally, the practical applicability of existing algorithms is limited, and their performance under real-world conditions requires further investigation. To address these challenges, this paper introduces SCR-Net, a water body identification model designed for multi-spectral remote sensing images. SCR-Net employs a dual-channel encoding–decoding mechanism and alters the number of channels used for reading image data, enhancing feature learning for lakes while focusing on extracting information about the water body target locations, thereby ensuring accurate segmentation. Trained on multi-spectral remote sensing images, the model leverages the unique spectral properties of these images to improve segmentation accuracy. Extensive validation on two datasets demonstrates that SCR-Net outperforms state-of-the-art models in terms of segmentation accuracy. Based on the validation using this dataset, Daihai Lake in Inner Mongolia was additionally selected as a case study to calculate the lake area, providing valuable insights for interdisciplinary research in ecological environment monitoring and remote sensing image processing.

## 1. Introduction

Lakes play a key role in regional water cycles, with their water storage capacity helping to regulate temperature, humidity, water cycles, and environmental purification processes [1]. However, due to unsustainable exploitation, many lakes have shrunk or even disappeared, leading to changes in organic matter levels within lake ecosystems and significant harm to the local environment [2,3]. To address this, environmental conservation professionals utilize remote sensing techniques to analyze imagery and quantify lake surface areas. These data are essential for monitoring ecological changes and supporting water resource management strategies aimed at maintaining the balance of lake ecosystems [4,5].

Water bodies exhibit distinct spectral characteristics compared to other objects, and by using specific combinations of wavebands, their boundaries can be accurately delineated. Traditional methods for measuring lake area typically involve analyzing high-spectral imagery data with geographic information software, distinguishing water bodies from background areas based on various waveband combinations. Common remote sensing techniques include the Normalized Difference Water Index (NDWI) and spectral index methods [6,7,8,9]. However, these approaches require complex processing and interpretation of remote sensing imagery, with segmentation thresholds influenced by the spectral characteristics of different objects. Various objects (e.g., water bodies, forests, and buildings) display unique spectral properties, and the reflective properties of the same object can vary across different wavelength bands [10,11]. The complex mathematical principles underlying traditional methods limit their generalization capabilities and often hinder accurate detection of target features, thereby affecting the precision of the final results. Advances in computer vision have enabled more effective segmentation of remote sensing images through deep learning algorithms, facilitating accurate identification of target features [6]. Unlike spectral band interpretation, computer vision achieves superior segmentation accuracy by training models on diverse algorithms. These approaches focus on extracting spatial and structural characteristics from images, such as shape, texture, edges, and contextual data. By incorporating spatial information, computer vision techniques enable more intricate feature extraction and analysis compared to traditional methods. Deep learning has become increasingly prominent in computer vision [12,13], with notable models such as U-Net, DeepLabV3+, and Vision Transformer (ViT) [14,15,16].

Sentinel-2 multi-spectral imagery serves as the primary data source. This study involves preprocessing remote sensing images of lakes in the Qinghai Plateau, China, and generating multi-spectral data for model training. A dual-channel encoder model is proposed to enhance water identification, and its performance is compared with existing models for validation. Daihai Lake in Inner Mongolia is selected as a case study to estimate the lake’s surface area. An analysis of factors contributing to changes in lake area between 2019 and 2023 is also conducted. This approach integrates deep learning technology with ecological studies. The main purposes are as follows:

(1) Developing a multi-spectral dataset for high-altitude lakes involves selecting remote sensing imagery that is suitable for generating four-band image data by combining the visible light (RGB) and near-infrared (NIR) spectral bands for segmentation. We use the blue, green, red, and infrared bands as the primary spectral components for training our water identification model. By leveraging the unique multi-spectral characteristics of remote sensing images, it addresses the limitations of feature extraction from the training data sources, thereby improving the segmentation accuracy of lake regions.
(2) Based on deep learning techniques, we designed the dual-branch SCR-Net (Swin-Convolution-Res-Net) model and applied it to the segmentation of water body area. The original Swin Transformer model has been enhanced by replacing its self-attention feature extraction mechanism with a linear attention ConvFormer and incorporating a residual structure to improve feature extraction. To evaluate the performance of the proposed model, comparative experiments were conducted with classic deep learning-based semantic segmentation models, such as DeepLabV3+ and Swin-Unet [17,18,19].
(3) Most current research on lake segmentation algorithms for multi-spectral imagery has been conducted on public datasets [20], which limits their practical application [21]. Therefore, this study uses Daihai Lake as a case study to apply the model, utilizing multi-spectral imagery to calculate the lake area within the Daihai watershed in Inner Mongolia. This approach provides valuable insights for frontline ecological workers to understand trends in lake area changes and fills a gap in integrating deep learning models with lake ecological restoration efforts.

## 2. Relate Work

### 2.1. Semantic Segmentation of Remote Sensing Images

Deep learning models play a crucial role in the interpretation of remote sensing images, enabling the segmentation of target objects through diverse model architectures and data types. These methods have been extensively applied to tasks such as image classification [22], object detection, and semantic segmentation. One prevalent approach involves constructing models using convolutional neural networks (CNNs), where the convolutional architecture progressively extracts and fuses spatial and semantic features via operations like convolution, pooling, and upsampling, ultimately achieving pixel-wise classification. However, the convolution process can lead to a loss of spatial details, thereby compromising pixel-level classification accuracy. In recent years, Vision Transformers (ViTs) have emerged as an alternative for semantic segmentation. ViT leverages self-attention mechanisms to effectively capture global dependencies within images, enhancing performance in tasks such as water body and land cover segmentation in remote sensing imagery. Despite these advantages, the high computational complexity and substantial data requirements of ViT present ongoing challenges for its practical application in remote sensing [23].

To address the aforementioned challenges, we implemented a dual-faceted approach focusing on the composition of remote sensing image data and the model’s feature-learning capabilities. First, we capitalized on the unique characteristics of multi-spectral remote sensing data by leveraging spectral variations associated with the light reflection and absorption properties of different objects. This approach enhanced the spectral representation of water bodies, thereby significantly improving the image feature-learning process. Second, we developed a dual-channel encoding strategy to effectively utilize multi-spectral images, which bolstered the model’s feature extraction capabilities. A dedicated multi-spectral dataset was constructed to train the lake segmentation model, which was subsequently employed to segment water bodies within the study area. To assess the model’s generalization performance, the GID (GF-2 Image Dataset) [24] was utilized. Finally, based on image resolution, we conducted a statistical analysis of pixel points corresponding to water bodies to accurately calculate the lake area of Daihai.

### 2.2. The Classic Water Identification Methods for Remote Sensing Images

There is a limited body of research that integrates multi-spectral images with deep learning algorithms. Most existing approaches primarily rely on visible light imagery for training datasets, which leads to suboptimal classification performance for specific targets due to the absence of comprehensive spectral information. Furthermore, the under-utilization of water identification algorithms in practical settings has emerged as a pertinent concern [10,21,25].

Currently, the primary research focus lies in the selection of diverse spectral data and algorithmic design for multi-spectral water identification. In 2021, Yuan et al. [25] proposed a multi-channel water detection network capable of feature learning from RGB, NIR, and SWIR bands. Zhong et al. [26] developed a model that enhances the segmentation capability of water bodies under varying lighting conditions, demonstrating superior performance in comparison to traditional CNN models for water identification tasks. In 2022, Tao et al. [27] devised MSNet, an algorithm for extracting water from multi-spectral remote sensing images. Leveraging the high absorption properties of water in the near-infrared band to differentiate it from vegetation and other objects, its exceptional performance was validated on the GID and Potsdam datasets. Zhang et al. utilized the SE (Squeeze-and-Excitation) [28] attention module within a residual network to extract water bodies from remote sensing images [29]. In the same year, Wang [30] introduced the SADS-Net model for the extraction of water bodies from high-resolution remote sensing images. The model acquired features from four-band data by enhancing the learning of multi-scale context information. Yu [31] investigated a novel multi-branch attention network model based on remote sensing images, extracting different semantic features at various levels and incorporating an attention mechanism to capture spatial and channel information in high-resolution remote sensing images, thereby improving segmentation accuracy. In 2023, Ma et al. [32] leveraged the state-of-the-art Swin Transformer algorithm for the analysis of multi-spectral remote sensing images. They introduced the WISTE model for water identification, harnessing the superior capabilities of the Swin Transformer architecture in computer vision tasks to achieve precise delineation of water bodies within multi-spectral data. Zhang et al. [33] devised a U-net-based model to extract water bodies from remote sensing imagery, facilitating comprehensive feature learning across individual spectral bands. Different from the above-mentioned research methods, we introduce a novel semantic segmentation network named SCR-Net. This network utilizes a dual-channel architecture with ConvFormer and residual encoding in its backbone and incorporates the GAM spatial-channel attention mechanism to mitigate information dispersion and enhance global interaction, thereby significantly improving the segmentation performance of the neural network.

### 2.3. Study Area Overview

**Figure 1:**
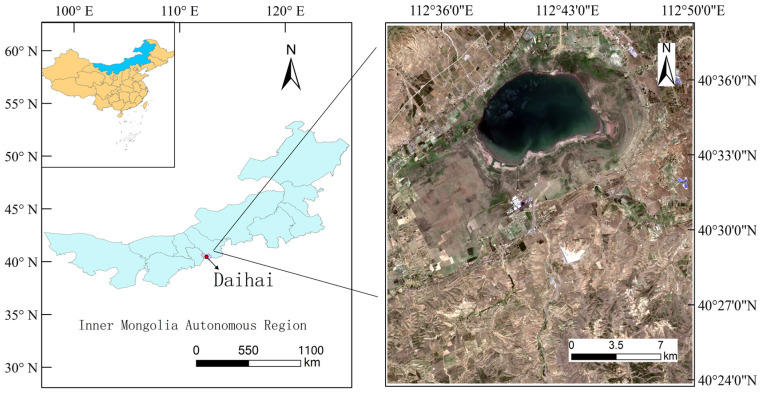
Location map of the study area; the image was acquired from the data of the Sentinel-2 satellite sensor on 18 July 2024, with a cloud cover of 2.52%.

Daihai is located in Ulanqab, Inner Mongolia Autonomous Region, China, south of the eastern segment of the Yinshan Mountains in northern Inner Mongolia. It is a typical endorheic, slightly saline lake, with geographic coordinates ranging from 112°33′ E to 112°47′ E and 40°30′ N to 40°37′ N, as shown in Figure 1. The Daihai Nature Reserve is surrounded by mountains, forming a classic closed inland watershed [34].

Daihai Lake is situated at the lowest point of the Daihai Basin and is primarily fed by surface runoff, groundwater, and precipitation. There are no outflow rivers; instead, water loss occurs through evaporation from the lake’s surface. Although several rivers flow into Daihai, none are permanent, yet they contribute over 50% of the total inflow to the lake. The water level in Daihai Lake remains relatively stable throughout the year, typically fluctuating by less than 1 m annually. The northern rivers are short and fast-flowing, with minimal industrial discharge entering the lake through inlet streams. However, agricultural non-point source pollution continues to be a significant concern for Daihai [35].

According to data from the Natural Resources Bureau of Inner Mongolia Autonomous Region, China, the surface area of Daihai Lake has been steadily decreasing over the past 60 years. From 1960 to 1971, the surface area remained relatively stable, averaging 158.81 km^2^. Between 1972 and 1982, it slightly decreased to an average of 147.67 km^2^. Since 1983, with few exceptions, the surface area sharply declined from 139.51 km^2^ to just 53.9 km^2^ in 2019. Concurrently, the water storage capacity has significantly decreased, from 1.27 billion cubic meters in the early 1960s to only 0.174 billion cubic meters in 2019—an alarming reduction of approximately 86%. This decline highlights the urgent need for the development of new algorithms capable of accurately calculating the lake area in the Daihai Basin using remote sensing images, which is essential for effective monitoring and record-keeping—a critical step for ecological and environmental protection in the region.

## 3. Method

Despite the use of numerous models for water identification in remote sensing images, their data formats and model training processes differ significantly from ours. Our model consists of three main components: the ConvFormer branch encoder, the residual structure branch encoder, and the feature fusion upsampling decoder. Initially, the processed multi-spectral data are fed into the model and passed through both the ConvFormer branch encoder and the residual structure branch encoder to learn image features. These features are then integrated into the upsampling decoder, which progressively restores the resolution and generates the final predicted image.

### 3.1. Overview of the SCR-Net

This section presents the SCR-Net model for multi-spectral image water identification, featuring an architecture that includes an encoder and a decoder within a network framework. The encoder employs a dual-branch structure to extract image features, enhancing the model’s feature-learning capacity and reducing spatial feature loss caused by image downsampling and resolution reduction.

**Figure 2:**
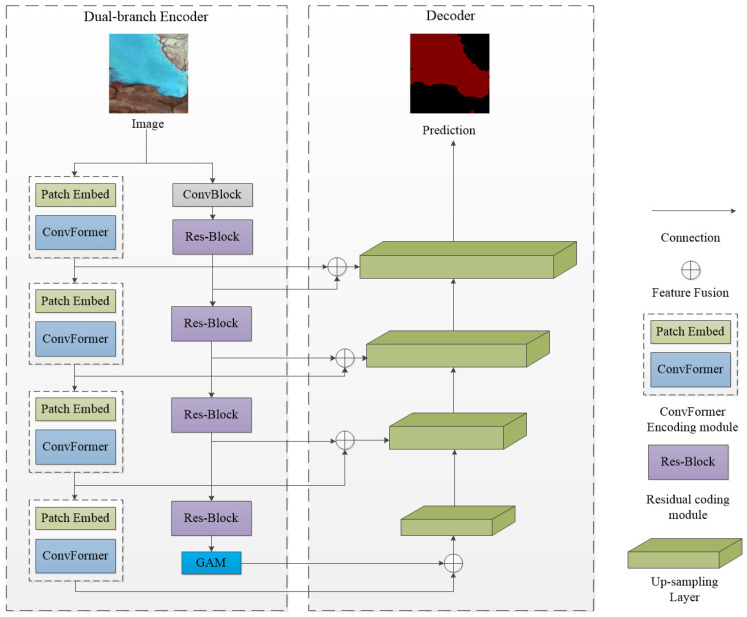
Overall structure of the proposed SCR-Net method, the left side depicting the encoder structure and the right side showing the decoder structure.

The ConvFormer module is designed to extract and learn image features through convolutional operations, emulating the concept of self-attention in Transformers by replacing attention matrix computations with convolutional layers. Additionally, the ResNet-50 model is employed for downsampling and feature learning, with a global attention module incorporated to enhance the aggregation of global information. The decoder performs feature fusion and upsampling operations, progressively integrating image features and restoring resolution through convolutional and multi-scale fusion modules. Ultimately, this results in the final prediction for image segmentation, as shown in Figure 2.

### 3.2. ConvFormer Encoder Architecture

**Figure 3:**
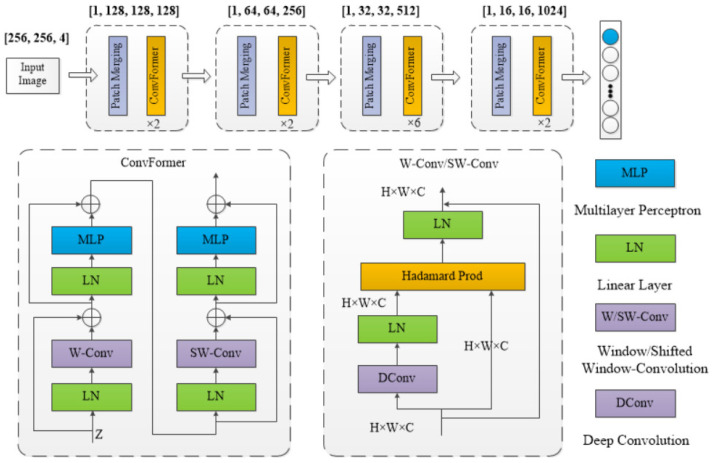
The overall ConvFormer encoding module; the top section presents the comprehensive structure, while the left side below depicts the structure of the ConvFormer module, and the right side illustrates the W-Conv or SW-Conv structures within the ConvFormer module.

In recent years, the Swin Transformer has shown significant advantages in semantic segmentation tasks. Unlike traditional convolutional neural networks (CNNs), Swin Transformer models global information through self-attention mechanisms and window partitioning strategies, capturing long-range spatial correlations and effectively learning image features for visual tasks. Consequently, we adopt a window-based learning strategy to extract image features using convolutional methods. The ConvFormer architecture was developed based on the Conv2Former structure [36], as illustrated in Figure 3. In this framework, the encoder initially processes the input image through a Patch Merging layer, employing a Transformer-like structure to perform segmentation and downsampling. For example, in the first stage, the original image of size [256, 256, 4] is transformed to [64, 64, 64], followed by passing through a linear layer to obtain the patch image of size [64, 64, C] (C denotes the number of channels; dimensions for each layer are sequentially 128, 256, 512, and 1024).

The data within each patch are processed using a convolutional structure to compute their self-attention score within the window, replacing the computation of the q, k, and v matrices in the original framework. This approach enables feature learning for 4-channel input images and aligns with the sliding-window strategy used in the Swin Transformer, ensuring effective interaction among different sub-images and enhancing the collection of global feature information. The design of the convolutional attention module is inspired by the self-attention mechanism of the Transformer Block, utilizing deep convolution to compute image attention weights. The formula is as follows:

(1) Z=A+X

The output A is obtained by modulating the linear features of the input data through the Hadamard product. Z denotes the image attention weight. Here, X represents the original input image data. The definition of the Hadamard product is as follows:

(2) $${\left(A\circ B\right)}_{ij}=A_{ij}\times B_{ij}$$

The indices i and j denote the elements in row i and column j of matrices A and B, respectively. The input image has dimensions (H,W,C) and is initially processed through a linear layer to facilitate subsequent operations on two pathways. On the left side of the diagram, a depthwise convolution is employed to conduct grouped convolutions on individual channels of the image data using a 7 × 7 convolution kernel with a padding size of 3, as depicted in Equation (3).

(3) $$D=W_{1}(C\mathrm{on}v_{7\times 7}(X))$$

In this equation, $W_{1}$ represents a linear operation on the input X to ensure that the output maintains consistent dimensions. The designed deep convolution is capable of capturing pixels within a 7 × 7 square region centered at (H,W), enabling the extraction of a broader range of local information and facilitating the model’s acquisition of more spatial features. In the right path, the input is preserved, and modulation of its linear features is achieved using Hadamard product, as illustrated in Equation (4):

(4) $$A=D\circ W_{2}(X)$$

In this architecture, $W_{2}$ denotes a linear transformation applied to the input of the second pathway, ensuring dimensional alignment for attention computation. The resultant matrix from deep convolution is utilized for feature weighting, and subsequently, a linear layer is employed to modulate data channel dimensions in constructing a residual structure to enhance learning efficiency. This architectural design streamlines model complexity and mitigates the computational burden associated with parameter multiplication arising from self-attention matrix generation.

### 3.3. Residual Encoding Module

**Figure 4:**
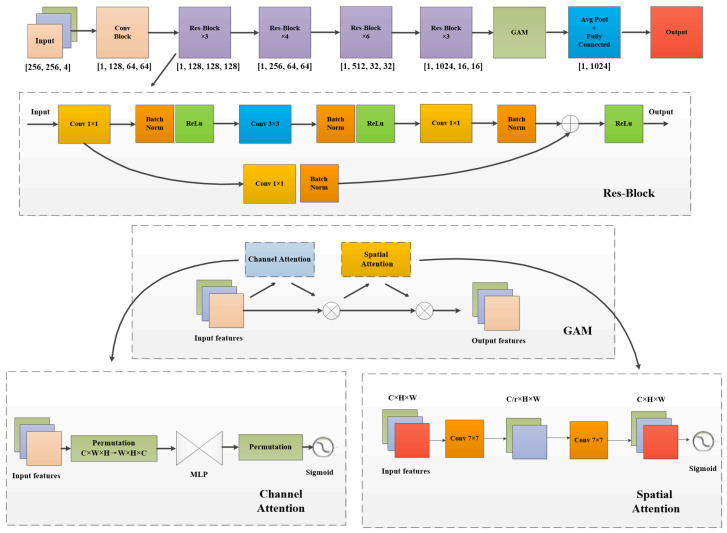
The overall architecture of the Residual Coding Module comprises the structure of the Res-block, the architecture of the GAM module, and the spatial attention and channel attention mechanisms within the GAM module.

Additionally, after acquiring image features through the ConvFormer branch, we introduced a novel auxiliary branch to enhance the model’s local segmentation capability and precision. This auxiliary branch integrates a ResNet-50 [37] network with a global attention module, forming the structure illustrated in Figure 4. The process begins with the input image, which passes through the Res-Blocks and GAM modules for progressive feature extraction, ultimately outputting the image features via a fully connected layer. During model training, an increase in network depth leads to more comprehensive feature extraction; however, this depth escalation may also result in a decrease in model accuracy [38]. Nevertheless, ResNet-50 effectively mitigates issues related to gradient explosion and vanishing gradients during training through its residual structure. As a result, we employ ResNet-50 to enhance feature granularity across different semantic scales. To further improve the performance of the model, a global attention module was incorporated into the final layer of ResNet-50 to strengthen the model’s ability to perceive global contextual information. The global attention module [39] specifically focuses on features across different channel dimensions, amplifying spatial feature information to identify important regions and objects within the image, thereby enriching feature details and enhancing semantic understanding. Assuming an initial input feature $F_{1}\in R^{H\times W\times C}$, the expressions for output $F_{2}$ from channel attention and output $F_{3}$ from spatial attention are as follows:

(5) $$F_{3}=M_{S}(M_{C}(F_{1})\cdot F_{1})\cdot F_{2}$$

One of them, $M_{c}$, represents the channel attention structure depicted in the figure, which enhances the inter-channel spatial dependency of diverse information dimensions through a dual-layer MLP. $M_{s}$ embodies the spatial attention mechanism by integrating spatial information using two 7 × 7 convolutional layers and reducing channel count by a factor of r=4 to mitigate computational overhead and parameter volume. Ultimately, the Sigmoid function is employed to focus on salient features within the image.

### 3.4. Feature Fusion and Upsampling

**Figure 5:**
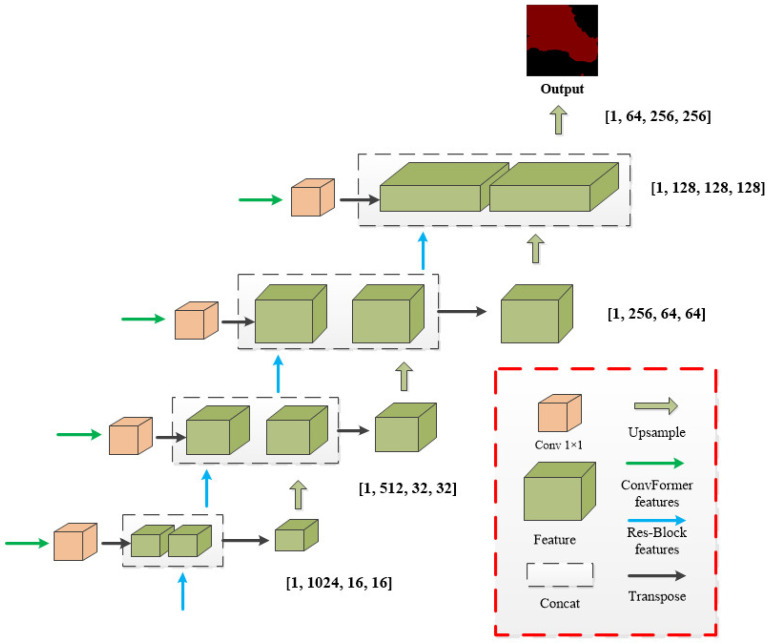
The structure of the Upsample module includes the convolutional layers and a schematic illustration of the feature fusion process.

Following feature extraction by the encoded model, we developed a feature fusion and upsampling module to restore image resolution. As shown in Figure 5, the features extracted by the ConvFormer branch are reshaped to match the size of the feature map from the residual feature extraction module. These features are then integrated and up-sampled using a concatenation operation to restore the image resolution. In dual-channel model research, common approaches include the integration of attention modules, the establishment of convergence relations, and feature mapping. However, these methods often lead to high computational complexity. Our design simplifies the feature adjustment and fusion process, preserves the integrity of semantic segmentation with the fewest parameters, and enhances model segmentation efficiency.

### 3.5. Evaluation Indicators

In semantic segmentation tasks, the model’s performance is primarily evaluated using metrics such as mean intersection over union (*mIoU*), recall, pixel accuracy, and *F*1-*score*. These metrics are calculated based on True Positives (*TPs*), True Negatives (*TNs*), False Positives (*FPs*), and False Negatives (*FNs*). Specifically, *TP* refers to the number of pixels where both the prediction and label correspond to water, *TN* indicates the number of pixels where both the prediction and label correspond to background, *FP* represents the number of pixels where the prediction is water but the label is background, and *FN* denotes the number of pixels where the prediction is background but the label is water. The formulas for these evaluation metrics are as follows:

(6) $$IoU=\frac{TP}{FN+TP+FP}$$

(7) $$PA=\frac{TP}{TP+FP}$$

(8) $$Accuracy=\frac{\left(TP+TN\right)}{\left(TP+TN+FP+FN\right)}$$

(9) $$F1\text{-}score=\frac{2TP}{2TP+FP+FN}$$

(10) $$Recall=\frac{TP}{TP+FN}$$

The intersection over union (*IoU*) metric represents the average ratio of intersection over union between the predicted and true values for water and background. Pixel accuracy (*PA*) denotes the proportion of correctly classified water pixels, while accuracy indicates the overall proportion of correctly classified water and background pixels. The *F*1-*score* balances precision and recall, serving as their harmonic mean. Recall reflects the proportion of correctly identified water pixels out of all actual water pixels. These evaluation metrics were selected due to their widespread use in current research on semantic segmentation tasks [26,40,41] and are commonly employed to assess model segmentation performance.

## 4. Experiments and Results

This section begins with an overview of the dataset preparation process, followed by an introduction to the evaluation metrics and an analysis of the training results. It also discusses the training environment and the selection of hyperparameters. Additionally, it introduces various commonly used semantic segmentation evaluation metrics to assess the performance of different models. Finally, it compares and analyzes the training results of different models and conducts ablation studies to evaluate the importance of each component within the models.

### 4.1. Overview of the Dataset

This study aims to explore the potential of integrating multi-spectral high-altitude lake datasets with GID multi-spectral datasets for research purposes. Specifically, we curate a multi-spectral high-altitude lake dataset to facilitate the pre-training of deep learning models. Additionally, we conduct a comparative experiment using GID multi-spectral remote sensing imagery to validate the model’s performance.

#### 4.1.1. Overview of Multi-Spectral High-Altitude Lake Model Training Datasets

**Figure 6:**
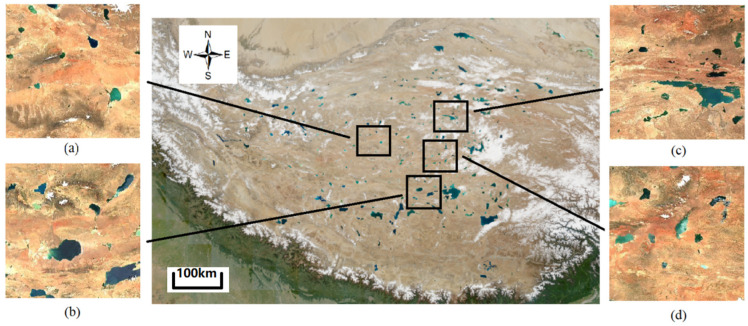
Multi-spectral lake dataset of the Tibetan Plateau images: (**a**) the acquisition time is 29 September 2023, with a cloud cover of 7.4%; (**b**) the acquisition time is 21 July 2023, with a cloud cover of 2.2%; (**c**) the acquisition time is 21 September 2023, with a cloud cover of 0.1%; (**d**) the acquisition time is 21 July 2023, with a cloud cover of 5.1%.

This study aims to employ deep learning algorithms to accurately estimate the lake area in the Daihai watershed using multi-spectral remote sensing images. Prior to applying the deep learning model, extensive pre-training is required to obtain optimal weight information. For this purpose, we collected remote sensing images of lake areas in the Qinghai–Tibet Plateau region of China to construct a multi-spectral dataset (Figure 6), sourced from the European Space Agency “https://dataspace.copernicus.eu (accessed on 21 January 2025)”. The images represent randomly selected samples from the Qinghai–Tibet Plateau region in China. The B2, B3, B4, and B8 bands of the original satellite images were combined specifically for the construction of the lake dataset. Both the Qinghai–Tibet Plateau and the Daihai region are part of the plateau lake system, which includes over 1000 lakes larger than 1 km^2^, characterized by a high density and abundance of lakes. This makes the region an ideal choice for the training dataset in this study.

The Sentinel-2 satellite is equipped with a multi-spectral sensor that captures images across 13 spectral bands, with resolutions ranging from 10 m to 60 m. In this study, L2A-level data from the Sentinel-2 satellite was selected for analysis. It represents one of the current options for high-quality datasets. The B2, B3, B4, and B8 bands from the original satellite images were combined, cropped, and annotated to create the dataset. In selecting spectral bands for this study, two factors were considered. First, the spectral characteristics of the target for segmentation were taken into account. Since the primary objective is to identify water bodies, and considering their high absorption and low reflection in the near-infrared spectral band [42], the infrared band was incorporated into the multi-spectral component of the dataset.

However, the Sentinel-2 satellite provides multiple infrared bands, which, as demonstrated in some studies, can lead to an increased number of training parameters without a significant improvement in training effectiveness [43]. Additionally, the resolution of the imagery data also affects segmentation outcomes [44,45]. Therefore, our selection criteria prioritize minimizing the number of bands to reduce training parameters while utilizing high-resolution imagery. Ultimately, we chose a dataset consisting of blue (B2), green (B3), red (B4), and near-infrared (B8) bands, all with a 10 m resolution.

**Figure 7:**
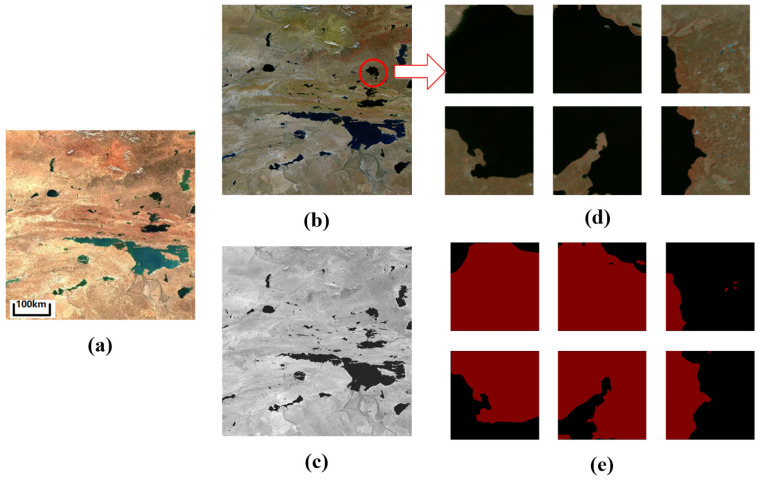
Multi-spectral dataset of plateau lakes. (**a**) The original remote sensing image, which includes the blue (B2), green (B3), and red (B4) bands; (**b**) a composite image of the blue (B2), green (B3), red (B4), and near-infrared (B8) bands in TIFF format, displaying a false-color image synthesized from the blue (B2), green (B3), and near-infrared (B8) bands; (**c**) the infrared band image (B8). The cropped result of the false-color image is shown in (**d**), and (**e**) presents the corresponding label.

We constructed multi-spectral images using the natural light bands 2, 3, and 4, along with the near-infrared band 8, for the dataset. Due to the large size of each original remote sensing image, direct input into the model for training was not feasible. Therefore, the images were partitioned into 256 × 256 TIFF-format tiles, and data not meeting the training requirements were manually removed. Pixel-level annotations of the segmentation targets were performed using Labelme software (version 5.2.1, “https://github.com/labelmeai/labelme (accessed on 21 January 2025)”), resulting in a total of 2101 images with corresponding label data. Figure 7 elaborates on the data production process for the image presented in Figure 6c.

#### 4.1.2. GID Multi-Spectral Datasets

During the investigation of training deep learning models, it was observed that using a single dataset could lead to overfitting, thus hindering the model’s generalization ability. To assess the efficacy of our multi-spectral deep learning model, we also employed the GID multi-spectral dataset released by Wuhan University, China [39], for algorithmic comparison and validation. The GID dataset is a large-scale, high-resolution collection of 150 images captured by the Gaofen-2 satellite, each with dimensions of 7200 × 6800 pixels and a resolution of 0.8 m. Based on the original metadata of the GID dataset, each image encompasses an area of 506 square kilometers, with a spatial resolution of 3.125 m per pixel (width) and 3.309 m per pixel (height). These images, collected from urban areas in southern China, include data across the blue, green, red, and infrared bands. Unlike imagery from highland lakes, these images were acquired in urban regions, where variations in altitude, light intensity, landform characteristics, and satellite sensors have caused differences in pixel values between datasets. However, such disparities provide valuable insights for testing the model’s generalization performance and improving learning outcomes for water features under diverse conditions.

**Figure 8:**
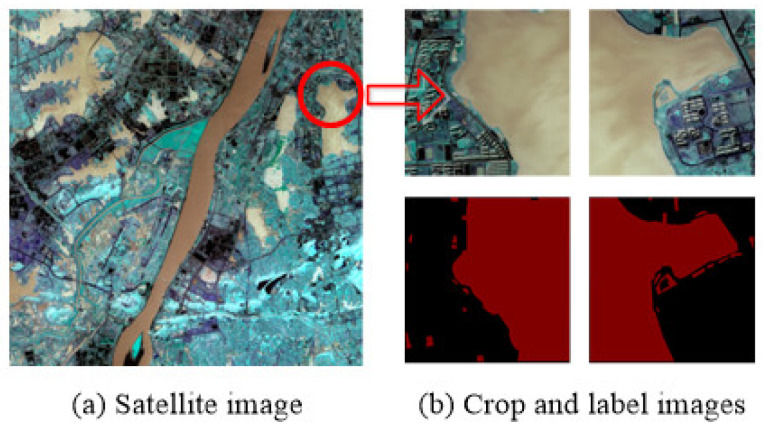
The training format of the GID dataset. (**a**) The original image chosen from the GID dataset; (**b**) the image data after cropping.

In this study, we selected 12 remote sensing images with a resolution of 7200 × 6800 pixels suitable for water identification tasks and cropped them to 512 × 512 pixels. The Labelme annotation tool was used to label the water body regions as “water” and all other non-water areas as “background”. After filtration, a total of 1548 RGB+NIR-band remote sensing images were obtained for water body training. The dataset format for model training is shown in Figure 8.

### 4.2. Training Environment and Hyperparameter Settings

All model training was conducted in a Windows 10 environment using Python 3.8 and the PyTorch 1.11.0+cu115 framework. The training process was carried out on an NVIDIA RTX 3090 GPU (24 GB memory). The dataset was randomly split into two parts, a training set and a test set, with a 7:3 allocation ratio. The model was trained using the training set and evaluated on the test set. For GPU memory allocation, the input image size for the multi-spectral dataset of high-altitude lakes is 256 × 256 × 4, while for the GID datasets, it is 512 × 512 × 4. The loss function used is BCELoss, and training employs the Adam optimizer “https://pytorch.org/docs/stable/optim.html (accessed on 21 January 2025)”. A batch size of 4 and an epoch count of 100 were chosen to ensure convergence of the model’s loss functions while preventing overfitting. The initial learning rate is set to 0.0001, with each model utilizing distinct pre-trained weights.

### 4.3. Results of Multi-Spectral High-Altitude Lake Dataset

**Figure 9:**
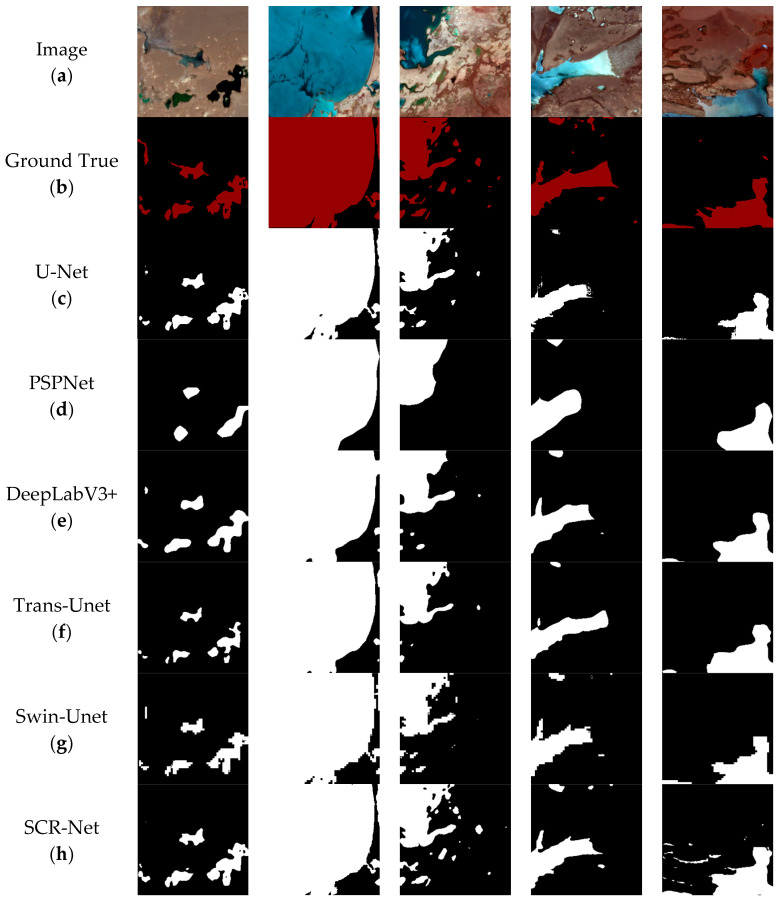
Visualization of predicted water identification results table. (**a**) The original image within the dataset; (**b**) the corresponding label image. (**c**–**h**) The predicted images generated by U-Net, PSPNet, DeepLabV3+, TransUnet, Swin-Unet, and SCR-Net models, respectively.

In this dataset, we conducted comparative experiments involving SCR-Net and other established semantic segmentation models, including U-Net, PSPNet, DeepLabV3+, TransUnet, and Swin-Unet. Figure 9 presents the visual representation of water identification predictions made by these models on the dataset. To clearly illustrate the discrepancies between the predicted map and the ground truth, water areas in the label map are marked in red, while the corresponding regions in the predicted images are shown in white. The average evaluation results for various models on the test set are summarized in Table 1, rounded to two decimal places. The SCR-Net model’s evaluation outcomes are highlighted in bold in Table 1.

**Figure 10:**
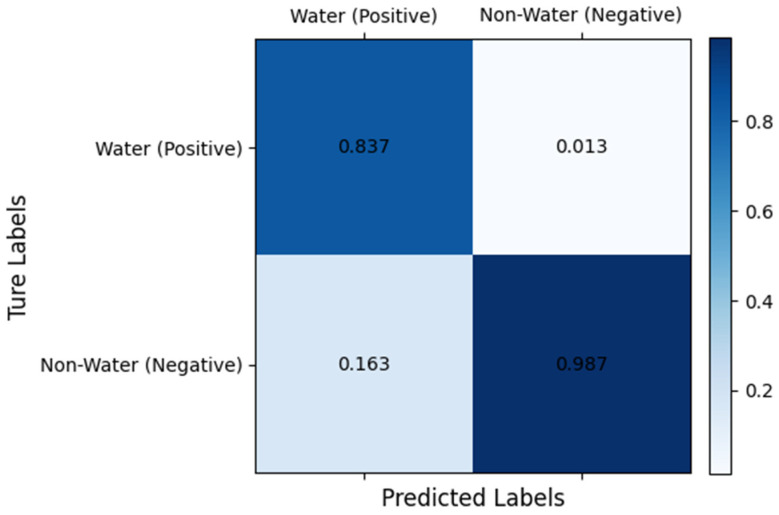
Confusion matrix of the multi-spectral lake dataset.

The visualization results of the highland lake dataset demonstrate that SCR-Net outperforms other models in accurately segmenting the water body area. When monitoring small water bodies, both PSPNet and DeepLabV3+ exhibit significant issues with missing predictions for most of these features. In contrast, the SCR-Net model shows minimal deviation from the ground truth, with fewer instances of misclassification and omission compared to other models when segmenting small lakes. In the context of large lake edge segmentation, the DeepLabV3+, TransUnet, and Swin-Unet models show suboptimal performance, particularly at the edges. The Swin-Unet model’s prediction map displays pixelated artifacts along the edges, which can be attributed to insufficient training data. Swin-Unet typically requires a large amount of training data to fully realize its potential. Our SCR-Net model, on the other hand, enhances feature diversity learning by focusing on capturing global contextual information through the ConvFormer branch and addressing gradient vanishing issues with the residual structure. This enables a more comprehensive extraction of water feature information and enhances local feature learning. Furthermore, Figure 10 presents the confusion matrix results for the validation set data. According to these results, the model achieves an 83.7% True Positive (TP) rate for correctly identifying water pixels, indicating that the majority of water areas are accurately segmented. The False Positive (FP) rate, representing misclassification of non-water pixels as water, is 16.3%. Additionally, the True Negative (TN) rate for correctly identifying background pixels is 98.7%, while the False Negative (FN) rate, where water pixels are incorrectly classified as background, is only 1.3%. These findings collectively demonstrate the model’s high accuracy in classifying both water and background regions. As a result, SCR-Net achieves higher interpretive accuracy, more precise edge segmentation, and improved pixel accuracy, even with limited datasets. When evaluating different models on this dataset, SCR-Net consistently achieved the highest scores across all metrics, demonstrating a marginal improvement over the second-best model in various evaluations and ultimately showcasing superior overall performance.

### 4.4. Results of GID Dataset

**Figure 11:**
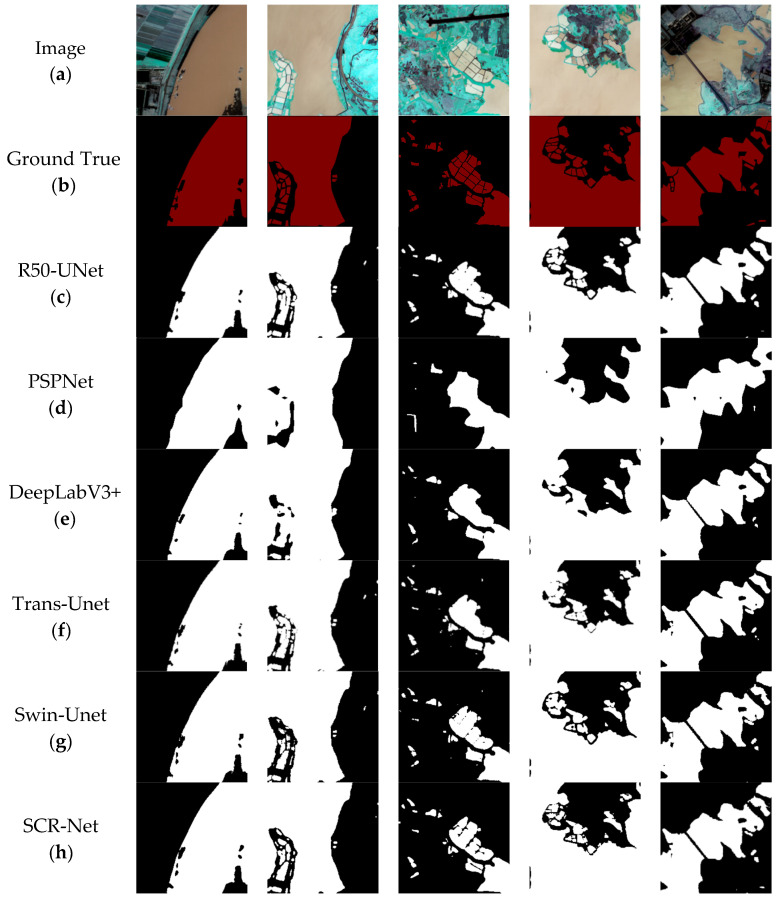
Visualization results of the GID dataset. (**a**) presents the original image, while (**b**) shows its corresponding label image. (**c**–**h**) depict the prediction results of U-Net, PSPNet, DeepLabV3+, TransUnet, Swin-Unet, and SCR-Net models, respectively.

In this section, we conduct experiments on the GID dataset to assess the performance of various models across different datasets. To enhance clarity, distinct colors are used to represent the true and predicted images in Figure 11. Our experiments on the GID dataset yielded consistent results, and upon comparing the six models, it was evident that the PSPNet model under-performed across all metrics. The R50-UNet and Trans-Unet models showed slightly lower efficacy compared to the SCR-Net model. The water body area in the GID dataset differs from that in the plateau lake dataset. Specifically, the GID dataset features a denser distribution of water bodies with more complex characteristics, which poses a greater challenge to model learning and results in smaller differences between the performance of various models. The models’ ability to segment the edges of water bodies within such dense areas is consequently limited. The dual-channel encoding structure employed in our model enhances its robustness and stability, enabling more accurate differentiation of semantic categories within complex environments and improving the delineation of water regions. The evaluation metrics are presented in Table 2.

**Figure 12:**
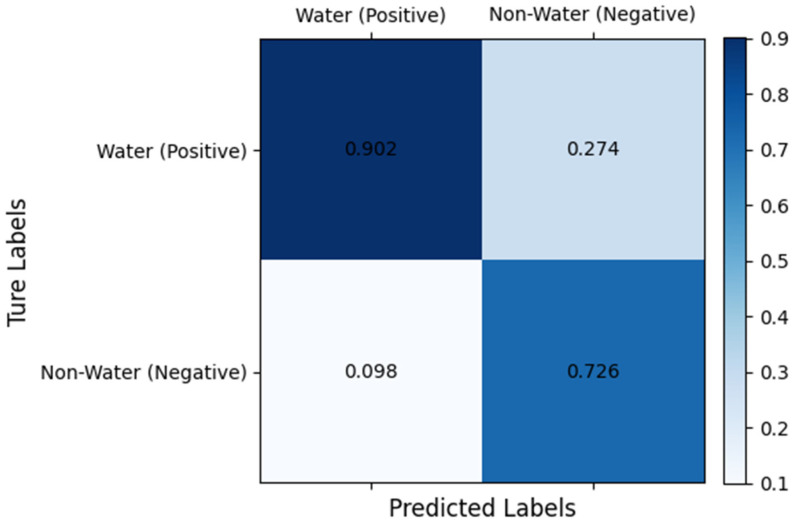
Confusion matrix results of the GID dataset.

After visualizing the results and evaluating the indicators across both datasets, we determined that our SCR-Net model exhibits superior feature-learning capabilities across various multi-spectral data types. This enables effective segmentation of water bodies, including both lakes and urban areas. Furthermore, the confusion matrix results in the Figure 12 for the GID dataset indicate that the model achieves a higher True Positive (TP) rate, as evidenced by the relatively high accuracy and precision metrics. This suggests that the model exhibits high accuracy in predicting water body areas, thereby demonstrating excellent overall performance in the water body segmentation task.

### 4.5. Ablation Experiment

In this section, we conduct ablation experiments [46] to assess the significance of each component within the model. The ablation studies were performed using the highland lake dataset. Specifically, we carried out two sets of experiments: first, we removed the ConvFormer branch structure to create a single encoder model based on ResNet-50; second, we excluded the ResNet-50 branch, configuring a single encoder model with ConvFormer and incorporating the GAM attention module for comparison. These ablation experiments were designed to validate the effectiveness of the dual-branch structure and the attention module and to analyze their individual contributions. The multi-spectral high-altitude lake dataset was used for the experiments presented in Table 3, where different model combinations were evaluated. Additionally, we generated both RGB images and original RGBN images from this dataset to demonstrate the effectiveness of the multi-spectral dataset, as shown in Table 4. Furthermore, we performed an ablation study on the model’s parameters, comparing the number of parameters for different algorithms after inputting RGB images. The detailed results of this analysis are provided in Table 5.

During the experiment, it was observed that the architecture of the single-branch encoder had a significant impact on the model’s performance, leading to decreased IoU, pixel accuracy, and recall rates, regardless of whether the ConvFormer or ResNet-50 branch structure was used. The influence of the ConvFormer branch on the model was found to be less effective than that of the ResNet-50 branch, which can be attributed to the integration of the GAM attention module into the ResNet-50 structure. This pattern was also reflected in the evaluation metrics. Furthermore, as shown in Table 4, training the model with RGB image data yielded less favorable results compared to using RGBN image data. The inclusion of near-infrared band data significantly improved water identification performance. Moreover, as shown in Table 5, we computed the parameters for three models by inputting three-channel 256 × 256 images. Based on the results, the parameter count of our model is higher than that of the Swin-Unet model due to its dual-channel structure. However, the parameter count of our model is still lower than that of the Trans-Unet model, while its segmentation performance surpasses that of both models. Therefore, our model demonstrates superior performance in the water identification task.

### 4.6. Determination of the Surface Area of Daihai Lake

After conducting the aforementioned experiments, it can be concluded that our SCR-Net model achieves superior accuracy in the water identification task, with a precision rate of 91.65% for detecting water body regions. In this study, satellite imagery data for the Daihai research area, spanning from 2019 to 2023, were acquired, as detailed in Table 6 below.

**Figure 13:**
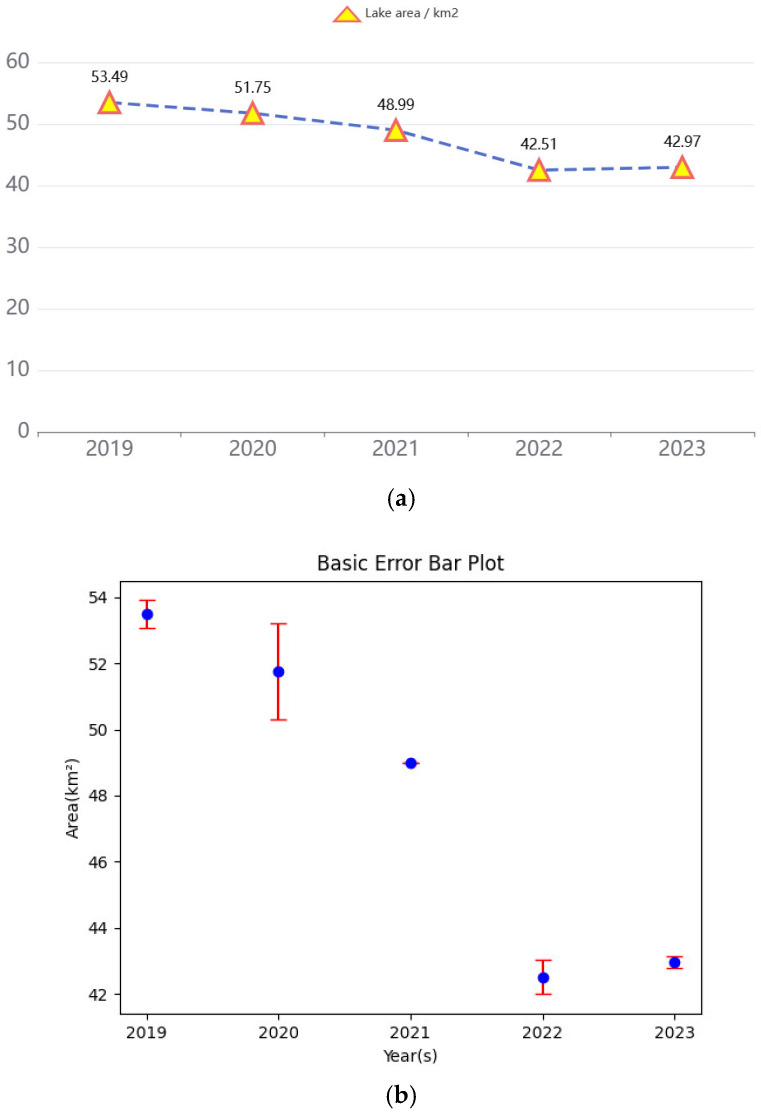
(**a**) The specific numerical prognoses regarding the area of Daihai Lake from 2019 to 2023, where (**b**) the blue line denotes our predicted area values, and the length of the red vertical line signifies the range of deviation from the actual value.

In selecting satellite images for Daihai Lake, we prioritize images with minimal cloud cover to reduce the impact of atmospheric interference on the lake area. Additionally, images from different years but corresponding months are chosen to ensure uniformity in the effects of precipitation and surface evaporation on Daihai Lake [47,48]. According to data from the National Meteorological Information Center of China “https://data.cma.cn/ (accessed on 21 January 2025)”, the administrative region where Daihai is located experienced the following monthly climate conditions from 2019 to 2023: average temperatures of −9.0 °C, 4.4 °C, −8.5 °C, −13.4 °C, and −11.4 °C; precipitation amounts of 1.6 mm, 13.4 mm, 0 mm, 0 mm, and 5.2 mm; and relative humidity levels of 49%, 41%, 46%, 44%, and 57%, respectively. Overall, the climate data indicate a stable period with no significant impact from extreme weather events. After pre-training, the SCR-Net model was employed to segment the lake area in the Daihai satellite imagery, and the number of pixel points representing water bodies in the segmented results was calculated. The actual area of the water body region was then determined based on pixel resolution and quantity. Finally, Figure 13a below presents the trend chart depicting changes in Daihai Lake’s area from 2019 to 2023.

We retrieved the records concerning the area of Daihai Lake spanning from 2019 to 2023 from the Inner Mongolia Institute of Geological Survey of China, compared them with the experimental results we procured, and constructed the error analysis chart presented in Figure 13b. The analysis of the data indicates that Daihai Lake in Inner Mongolia has continued to experience a reduction in its surface area over the past few years. The lake’s expanse has decreased from 53.49 km^2^ in 2019 to 42.97 km^2^ in 2023, representing an overall shrinkage of nearly 10 km^2^.

According to the accounts of relevant personnel in the Daihai watershed, Daihai Lake experiences high rates of evaporation and low inflow. Furthermore, the commissioning of Phase I of the Daihai Power Plant in 2006 introduced a jet-type unit that artificially impacts the lake by utilizing it as a direct current cooling pool. This has led to a sustained decrease in the water level and a substantial reduction in surface area. These findings align with our designed lake area partition model, affirming the practical applicability of the SCR-Net model.

## 5. Conclusions

This study introduces SCR-Net, a water identification model designed for multi-spectral remote sensing imagery. Its distinctive dual-branch encoding structure enables the specialized learning of different semantic features in each branch, facilitating the comprehensive integration of both global and local features. This approach significantly enhances the accuracy of pixel-level predictions for water identification. Furthermore, this research applies deep learning models to the field of ecological conservation, utilizing neural network algorithms to develop a lake segmentation model for multi-spectral remote sensing imagery. By effectively leveraging the unique multi-spectral characteristics of such imagery, the model is capable of accurately delineating both target and background information. Through the integration of multi-spectral data with deep learning algorithms, the model offers a promising new direction for the application of deep learning in lake ecological protection.

Future research will focus on leveraging datasets with enhanced spectral information to explore the impact of various spectral bands on segmentation performance. Further improvements to the model are anticipated, with strategies being developed to investigate different branch structures and architectures of deep learning models, aiming to optimize model performance for higher accuracy. Additionally, the comprehensive utilization of multi-spectral resources from remote sensing imagery will be explored for potential applications across a wide range of ecological domains.

## Figures and Tables

**Table 1:** Evaluation indicators for multi-spectral lake dataset.

Model	Accuracy	Precise	Recall	F1-Score	IoU
U-Net [14]	90.67%	79.50%	80.62%	78.34%	73.34%
PSPNet [15]	88.11%	61.06%	66.74%	61.92%	56.56%
Deeplabv3+ [16]	90.42%	75.35%	76.57%	74.53%	69.08%
Trans-Unet [46]	90.81%	79.05%	77.13%	76.64%	71.22%
Swin-Unet [47]	90.46%	74.59%	77.11%	74.35%	68.99%
**SCR-Net (Ours)**	**91.65%**	**83.74%**	**87.74%**	**83.52%**	**78.13%**

**Table 2:** Evaluation indicators for GID dataset.

Model	Accuracy	Precise	Recall	F1-Score	IoU
U-Net [14]	97.38%	87.53%	85.84%	86.12%	77.56%
PSPNet [15]	96.50%	77.33%	85.05%	78.83%	68.74%
Deeplabv3+ [16]	94.93%	67.04%	76.41%	68.57%	57.44%
Trans-Unet [46]	97.38%	85.93%	86.41%	85.43%	76.67%
Swin-Unet [47]	97.13%	84.14%	88.14%	84.39%	75.29%
**SCR-Net (Ours)**	**97.54%**	**90.16%**	**86.17%**	**87.64%**	**79.49%**

**Table 3:** Results of model structure ablation experiments.

Conv Former	ResNet-50	GAM	Accuracy	Precision	Recall	F1-Score	IoU
√			90.05%	82.38%	87.38%	83.84%	74.71%
	√	√	90.17%	83.14%	86.89%	83.89%	74.76%
√	√		90.14%	82.85%	87.20%	84.33%	75.24%
√	√	√	**91.65%**	**83.74%**	**87.74%**	**83.52%**	**78.13%**

**Table 4:** Results of spectral data fusion experiments with different datasets.

	Accuracy	Precision	Recall	F1-Score	IoU
RGB	90.58%	73.26%	78.05%	73.93%	68.32%
**RGB + Nir**	**91.65%**	**83.74%**	**87.74%**	**83.52%**	**78.13%**

**Table 5:** Comparative analysis of the quantity of model parameters among different models.

	Trans-Unet [46]	Swin-Unet [47]	SCR-Net (Ours)
Parameters (Millions)	73.06 M	34.24 M	67.02 M
Time (Seconds)	1.1662 s	0.2868 s	0.7021 s

**Table 6:** Satellite image data information.

Time	Cloud Cover
11 December 2019	0.4%
17 October 2020	1.1%
26 December 2021	19.4%
31 December 2022	0.6%
6 December 2023	0.1%

## Data Availability

The data used in this paper come from two sources. The multi-spectral lake remote sensing imagery dataset comes from the European Space Agency “https://dataspace.copernicus.eu (accessed on 21 January 2025)”, and the GID multi-spectral imagery dataset comes from the open dataset of Wuhan University “https://arxiv.org/abs/1807.05713v2 (accessed on 21 January 2025)”.
